# Adjusting Spectral Indices for Spectral Response Function Differences of Very High Spatial Resolution Sensors Simulated from Field Spectra

**DOI:** 10.3390/s150306221

**Published:** 2015-03-13

**Authors:** Sharon L. Cundill, Harald M. A. van der Werff, Mark van der Meijde

**Affiliations:** Faculty of Geo-Information Science and Earth Observation (ITC), University of Twente, PO Box 217, 7500 AE Enschede, The Netherlands; E-Mails: harald.vanderwerff@utwente.nl (H.M.A.W.); m.vandermeijde@utwente.nl (M.M.)

**Keywords:** cross-calibration, data continuity, dike, grass, levee, satellite, spectral index, spectral response function effect, spectral resolution, UAV, very high spatial resolution sensor

## Abstract

The use of data from multiple sensors is often required to ensure data coverage and continuity, but differences in the spectral characteristics of sensors result in spectral index values being different. This study investigates spectral response function effects on 48 spectral indices for cultivated grasslands using simulated data of 10 very high spatial resolution sensors, convolved from field reflectance spectra of a grass covered dike (with varying vegetation condition). Index values for 48 indices were calculated for original narrow-band spectra and convolved data sets, and then compared. The indices Difference Vegetation Index (DVI), Global Environmental Monitoring Index (GEMI), Enhanced Vegetation Index (EVI), Modified Soil-Adjusted Vegetation Index (MSAVI_2_) and Soil-Adjusted Vegetation Index (SAVI), which include the difference between the near-infrared and red bands, have values most similar to those of the original spectra across all 10 sensors (1:1 line mean _1:1_R^2^ > 0.960 and linear trend mean ccR^2^ > 0.997). Additionally, relationships between the indices’ values and two quality indicators for grass covered dikes were compared to those of the original spectra. For the soil moisture indicator, indices that ratio bands performed better across sensors than those that difference bands, while for the dike cover quality indicator, both the choice of bands and their formulation are important.

## 1. Introduction

Remote sensing data are widely used for vegetation, environmental, hazard and land process monitoring and assessment from local through to global scales. For monitoring, data from multiple sensors are often used in order to ensure coverage and continuity, particularly due to limitations of satellite revisit time [1], cloud cover [1,2] as well as satellite design life [3]. However, data obtained from different sensors are not directly comparable [1,4]. Differences in sensor specifications as well as scene-specific conditions (e.g., atmosphere, sun angle, *etc.*) affect measurements [3,5]. Cross-calibration between sensors is necessary for consistency and comparison of observations [1,6,7]. One of the main causes of differences in remote sensing data is the difference in spectral response functions (SRFs) between sensors [5,6,8,9,10]. The effect may be such as to mask subtle natural variability that is of interest [8,10].

It has been shown that SRF corrections are target (cover type) specific [6,11,12,13]. The cover type of cultivated grasslands, which includes cultivated pastures (used for fodder production, grazing, erosion protection, *etc.*) and turf grasses (such as lawns, parks and golf courses), cover substantial proportions of land surface. For example turf grass is estimated to cover some 1.9% (about 163,800 km^2^) of the continental United States [14] and permanent cultivated pastures cover about 18.4% (about 7664 km^2^) of The Netherlands [15], with thousands of kilometers of grass covered dikes [16,17]. Evaluation and monitoring of cultivated grasslands using remote sensing data are the subjects of numerous studies (e.g., [18,19,20]), including for inspection and monitoring of grass covered dikes and levees [21,22]. Consequently investigation of SRF effects for the cross-calibration of sensors for the cultivated grasslands cover type is required.

While some investigations of SRF effects for cross-calibration focus on comparing sensors at reflectance band level [6,7,23], many include spectral indices that provide information on plant biophysical parameters. In fact, Miura *et al.* [24] suggest that there may be an advantage to cross-calibrating at the index level rather than the reflectance band level, since indices generated from cross-calibrated reflectance bands may include bias errors that arise from not accounting for intra-sensor band to band correlations. Since the development of the attributed first vegetation index in 1969 [25], over a hundred different indices have been developed [21], addressing different aspects of improving vegetation characterization. Many aim at reducing the effects of extraneous influences (e.g., soil background, atmosphere) while others focus on specific chemical or structural components of vegetation (e.g., chlorophyll, water content). Various indices have been investigated for the evaluation and monitoring of turf grass [26,27], pastures [20] and a large number of spectral indices have been tested for use in the inspection of grass covered dikes [21]. Only a few studies have addressed the SRF effects for cross-calibration of indices other than the Normalized Difference Vegetation Index (NDVI). These studies, for 2–8 indices across up to 17 sensors [1,2,4,13,28], show that variations in the SRFs of different sensors result in significant differences in index values which must be corrected to avoid bias in observations.

Most cross-calibration related studies focus on low to medium spatial resolution sensors, such as AVHRR, MODIS and Landsat [3,4,6,9,10,13,24]. However, the small size of individual lawns and parks and the often long and narrow structure of dikes and golf courses dictate that remote sensing data of very high spatial resolution are required. A few studies have included higher spatial resolution instruments (e.g., IKONOS, QuickBird) amongst those studied [1,2,5,7,29,30,31], however SRF adjustment coefficients are dependent on the specific sensors studied and in particular, the sensor selected as reference. To the best of the authors’ knowledge, none have focused on very high spatial resolution sensors.

This research is conducted as part of on-going studies that are investigating the use of remote sensing data for the inspection of grass covered dikes. The initial research (see [21]) involved the testing of ground based remote sensing data for two dike inspection indicators. With the successful results of that research, a subsequent investigation is underway to test the use of imagery for the same application. However, since index values from different sensors are not directly comparable, this paper aims to investigate the effects of differing SRFs of various very high spatial resolution sensors on the cross-calibration of numerous spectral indices in the context of cultivated grasslands, that are typically found on dikes and levees that do not have a hardened cover.

## 2. Materials and Methods

**Figure 1:**
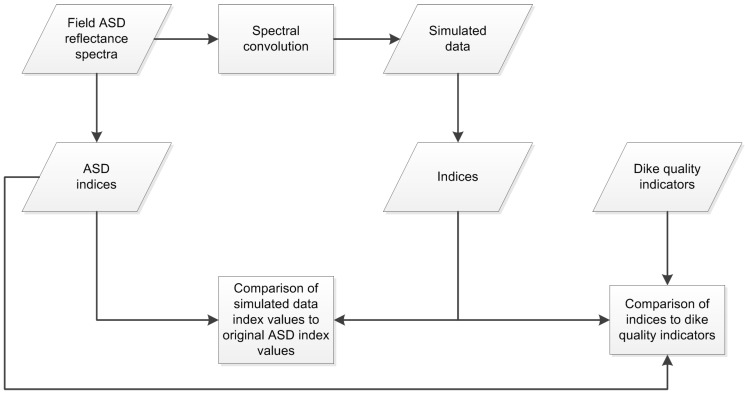
Summary workflow of the materials and methods used in this study.

A summary workflow of the materials and methods used in this study is given in Figure 1, with the details elaborated on in the remainder of this section.

### 2.1. Data

While some studies that address the effects of SRFs on cross-calibration between sensors make use of laboratory measurements (e.g., [32]), many make use data from airborne imagery (e.g., [4,30]), satellite imagery (e.g., [6,24]) or field measurements (e.g., [1,5]). Data from field measurements provide reflectance spectra of the vegetation canopy, practically free from atmospheric affects, and allow for precise measurement of specific locations and a range of conditions. The data used for this study are field measurements that were collected as part of a study testing ground based remote sensing data for two dike inspection indicators [21]. The data were collected for 54 locations on a grass covered dike in The Netherlands on 15 July 2010. The measurement grid consisted of six lines running along the dike spread over the geometric profile, with nine points per line at 5 m spacing. The grass on the dike is cultivated pasture and used for hay production as well as directly for grazing. Although the measurements were recorded during one of The Netherlands’ driest Julys on record, it should be noted that the soil moisture condition in part of the study area is thought to be influenced by long-term subsurface hydrological processes [21]. A wide range of grass conditions were thus present at the time of measurement, varying from extremely lush and green to substantial proportions of dry, dead grass and some bare soil, thereby making the data set representative of potential conditions for cultivated grasslands. The grass had not been recently mowed.

The data consists of three data sets: a set of reflectance spectra, a set of soil moisture measurements (referred to as the soil moisture indicator) and a set of grass cover assessments (referred to as the dike cover quality indicator). The ground-based reflectance spectra were obtained using an ASD FieldSpec Pro spectrometer (Analytical Spectral Devices, Boulder, CO, USA). The instrument has a wavelength range of 350–2500 nm, band widths of 2–3 nm, with a 1 nm sampling interval. An 8° foreoptic was used to constrain the field of view to the areas of interest and aid in the accurate placement of measurement locations. Each spectrum per location was an average of three spectra with sample counts of 25. A calibrated Spectralon^®^ panel was used as a white reference to calculate reflectance. Soil moisture was directly measured using a ThetaProbe Soil Moisture Sensor-ML2× (by Delta-T Devices Ltd., Cambridge, UK), averaging nine measurements per location. The quality of the dike covering was evaluated and classified into four classes. The evaluation criteria include grass density, canopy cover and the presence and quantity of standing litter (dead plant material), flotsam (floating debris), weeds, and bare soil. A comprehensive description of the data is available in Cundill *et al.* [21].

### 2.2. Spectral Convolution

**Figure 2:**
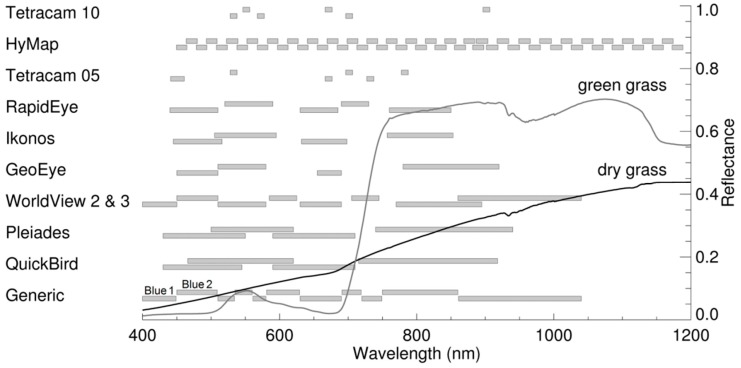
Band positions and widths for the sensors used in this study.

As cultivated grasslands are often relatively small or narrow, only sensors capable of a multispectral spatial resolution of 5 m or finer were considered. The selection includes both broad- and narrow-band sensors, hyper- and multi-spectral sensors and sensors that are mounted on satellite, airplane and unmanned aerial vehicle (UAV) platforms. Simulated data were generated by convolving the field ASD reflectance spectra to the spectral resolution of each sensor (Table 1, Figure 2) by means of their respective SRFs using the built in resampling functions in the ENVI 5.0 software (Exelis Visual Information Solutions, Inc., Boulder, CO, USA). Since most of these sensors do not have a shortwave infrared (SWIR) band, only bands in the visible and near-infrared are considered. The WorldView-3, HyMap and two Tetracam Mini-MCA sensors were not available as pre-defined functions in ENVI. Therefore, for the two Tetracam Mini-MCA sensors, the ASD data were convolved in ENVI using sensor specific SRFs which were obtained from the supplier. However, to the best of the authors’ knowledge, the SRFs for the HyMap and WorldView-3 sensors have not yet been published. Thus for these two sensors, ASD spectra were convolved using Gaussian shaped response functions based on the position and width of each band. The only variation between these simulated data sets is thus due to the sensor specific SRFs (or approximate SRFs for the HyMap and WorldView-3 sensors). This allows for the investigation of only the effects of differing SRFs since all other parameters (e.g., atmospheric conditions, sun and viewing geometry, spatial resolution, *etc.*) are identical between the data sets.

**Table 1:** Details of sensors used in this study (in order of maximum band width from narrowest to broadest).

Sensor (Abbreviation)	Spatial Resolution	Spectral Type	Spectral Resolution	Bands	Platform
ASD FieldSpec Pro spectrometer	Dependent on height of sensor (non-imaging)	hyper	narrow (2–3 nm)	2151 contiguous bands between 350–2500 nm	ground
Tetracam Mini-MCA (TC10) [33] *	10 s to 100 s mm (dependent on height of sensor)	multi	narrow (10 nm)	6 bands between 520–910 nm	UAV/airplane
HyMap [34]	Dependent on height of sensor	hyper	narrow (15–20 nm)	128 contiguous bands between 450–2500 nm	airplane
Tetracam Mini-MCA (TC05) [33] *	10 s to 100 s mm (dependent on height of sensor)	multi	narrow (10–20 nm)	6 bands between 430–790 nm	UAV/airplane
RapidEye [35]	5 m	multi	broad (40–90 nm)	5 bands between 440–850 nm	satellite
IKONOS [36]	3.2 m	multi	broad (66–96 nm)	4 bands between 445–853 nm	satellite
GeoEye-1 [36]	1.65 m	multi	broad (35–140 nm)	4 bands between 450–900 nm	satellite
WorldView-3 (WV3) [37]	1.24 m	multi	broad (40–180 nm)	8 bands between 400–1040 nm	satellite
WorldView-2 (WV2) [38]	2 m (resampled)	multi	broad (40–180 nm)	8 bands between 400–1040 nm	satellite
Pléiades-1 [39]	2 m	multi	broad (120–200 nm)	4 bands between 430–940 nm	satellite
QuickBird (QB) [40]	2.62 m	multi	broad (115–203 nm)	4 bands between 430–918 nm	satellite

* Note: the Tetracam Mini-MCA is a configurable spectroscope, using changeable filters which the user selects.

### 2.3. Indices

The selection of spectral indices tested in this study (Table S1) is based on over one hundred indices tested by Cundill *et al.* [21], but limited to those that are applicable to at least three of the sensors being investigated (Table 1). The selected 48 indices cover general vegetation indices, moisture indices, as well as specific compositional or structural indices. Many were developed for narrow-band data but those developed for broad-band data (as indicated in Table S1) have previously been applied in narrow-band form in various publications (e.g., [41,42,43]). Likewise, narrow-band indices have similarly been applied in broad-band form (e.g., [44,45]). The current study includes both broad- and narrow-band sensors, and thus indices are applied in both their broad- and narrow-band form. Since the SRFs of broad band and narrow band sensors are considerably different, the index values calculated from these sensors are also be expected to be different. However, it is sometimes necessary to compare data from these two extremes. In addition, a number of studies have investigated whether the broad- or narrow-band form of an index performs better for a parameter. For example, Elvidge and Chen [46] found that for seven indices investigated, the narrow-band form performed better for percent green cover and leaf area index (LAI) than the broad-band form of the same index. Similarly, Thenkabail *et al.* [47] show that the narrow-band form of various indices performed better for biomass and LAI. On the other hand, Broge and Leblanc [48] demonstrate in their study that the broad-band form of ten indices generally appear to be better at predicting LAI and canopy chlorophyll density. The results of the current study contribute to this discussion.

The equations for the indices in Table S1 are in the form for narrow-band hyperspectral data. If the specified wavelength was not available in the HyMap data set, then the band with the center wavelength closest to this wavelength was used. For the other data sets, wavelengths were grouped into generic bands (Figure 2). For each sensor or index, a specific band or wavelength was allocated to a generic band based on whether the center wavelength of the sensor band or index wavelength fell within the generic band’s wavelength range. Exceptions were made when a sensor had only one band in a particular region of the electromagnetic spectrum (e.g., only generic Blue 2). In these cases, the same reflectance values were allocated to the remaining generic band/s for that region of the electromagnetic spectrum (e.g., Blue 1). This was done in order to limit the effect of the arbitrary allocation of wavelength range to the generic bands which may have resulted in the invalidity of an index for a particular sensor. An index was considered not valid (indicated by NA) for a particular sensor when either the required wavelengths were not available, or when the index became equivalent to another index as a result of using broader bands.

### 2.4. Analysis

The analysis to investigate the effects of SRFs and performance of the indices from different sensors took two main forms. The first was to evaluate index values generated from convolved narrow- and broad-band data compared to the index values obtained from original narrow-band ASD data. The original narrow-band ASD data were selected as the reference data set for this study because the 2–3 nm band widths of the ASD sensor are an extreme case of narrow-band sensors against which the indices calculated from broader-band sensors could be tested, thereby testing the robustness of the indices across a wide range of spectral resolutions. The band widths of the other sensors range between 10 nm and 203 nm. In addition, narrow-band multispectral sensors, such as the Tetracam Mini-MCA, are becoming increasingly popular, especially for use on UAVs. These narrow-band sensors do not allow the averaging of wavelengths to simulate broad bands and so direct comparison of index values from narrow- and broad-band sensors is useful. Further, Yao *et al.* [49] point out a lack of cross-calibration studies that systematically compare narrow-band indices with broad-band indices. The other form of analysis was to compare the performance of indices from various sensors for an application, *i.e.*, inspection and monitoring of grass covered dikes. The use of the original ASD data as the reference data set also allows for comparison with the results from Cundill *et al.* [21].

#### 2.4.1. Comparison to Original ASD Index Values

To evaluate the effects of differing SRFs on indices between sensors, index values for simulated data were compared to those for original ASD data. Four measures were used to indicate the extent to which the simulated data deviate from the original ASD data, namely the slope and intercept of the linear trend line, and two variants of the coefficient of determination.

A perfect match between the index values for the original ASD data and those of the simulated data should render a linear trend line with a slope of 1 and intercept of 0, the so-called 1:1 line. The linear trend was computed for each of the simulated index data sets, and the slope and intercept for these trend lines were compared to the 1:1 line. Since the value of the intercept is relative to the values for a particular index, the intercept was normalized via division by the mean of the ASD index values.

The two variants of the coefficient of determination used in this study have the same basic equation structure but their definitions differ in that they use different data sets. The first variant of the coefficient of determination, the square of the correlation coefficient (Equation (1)), is calculated to test how the data fits the linear trend. To avoid confusion with the second variant of the coefficient of determination (calculated for the 1:1 line), the square of the correlation coefficient is denoted in this paper as ccR^2^. A perfect fit of the data to the trend line would render a ccR^2^ value of 1. Values can range between 0 and 1.

(1) $${\text{ccR}}^{2}=1-\;\frac{\sum_{\text{i}}\;{\left({\text{y}}_{\text{i}}-\;{\hat{\text{y}}}_{\text{i}}\right)}^{2}}{\sum_{\text{i}}{\left({\text{y}}_{\text{i}}\;-\;\bar{\text{y}}\right)}^{2}}$$

where ${\text{y}}_{\text{i}}$ denotes index values for the simulated data, ${\hat{\text{y}}}_{\text{i}}$ denotes modelled index values, and $\bar{\text{y}}$ denotes the mean of the index values for the simulated data.

The second variant of the coefficient of determination (denoted as _1:1_R^2^ in this paper to avoid confusion with the first variant of the coefficient of determination) provides information on the overall deviation of simulated index values from original ASD index values, relative to the 1:1 line (Equation (2)), similar to the Nash-Sutcliffe efficiency [50]. The simulated data have not been derived from a model fitting procedure, and thus values outside of the typical 0 to 1 range are possible since the test, in this case, is not using modeled values [51]. Ideally, _1:1_R^2^ should be 1, indicating that there is no difference between the simulated and original ASD index values. This is not expected, however, as the different SRFs of the various sensors will result in differing index values (as shown by the previous studies discussed in the Introduction).

(2) R21:1=1− ∑i (yASDi− ySIMi)2∑i(yASDi − y¯ASD)2

where $y_{ASD_{i}}$ denotes index values for the original ASD data, $y_{SIM_{i}}$ denotes index values for the simulated data, and ${\bar{y}}_{ASD}$ denotes the mean of the index values for the original ASD data.

For an objective overview of how an index performed across sensors, the mean for each measure per index was computed. This is straight-forward for the _1:1_R^2^ and ccR^2^. However, for the other measures where the values vary around a central value (1 for the slope and 0 for the intercept), adjustments needed to be done before the mean could be calculated. In the case of the intercepts, the absolute values (|intercept|) were used for the calculation of the mean. For the slopes, since the values vary around 1, the mean was computed from the absolute values of slope minus 1 (|slope − 1|).

#### 2.4.2. Correlation to Dike Quality Indicators

In a previous study [21], the relationships between index values from the original ASD data and two quality indicators for grass covered dikes were investigated. Now, to evaluate how the indices from the simulated data for various sensors perform for an application, the relationships between their index values and the two quality indicators for grass covered dikes are explored. The two quality indicators are soil moisture and the dike cover quality. Although the quality indicators are specifically defined here in the context of dikes, both soil moisture and condition of grass cover are the subject of many remote sensing studies relating to cultivated grasslands (e.g., [19,20,26,27]). The correlation coefficients between each index (for each sensor) and each quality indicator were computed, with the Pearson correlation coefficient used for the soil moisture quality indicator and the Spearman correlation coefficient used for the ordinal dike cover quality indicator. As for the previous measures, the mean for each correlation coefficient was computed per index, using their absolute values (|r|).

## 3. Results and Discussion

### 3.1. Comparison to Original ASD Index Values

**Figure 3:**
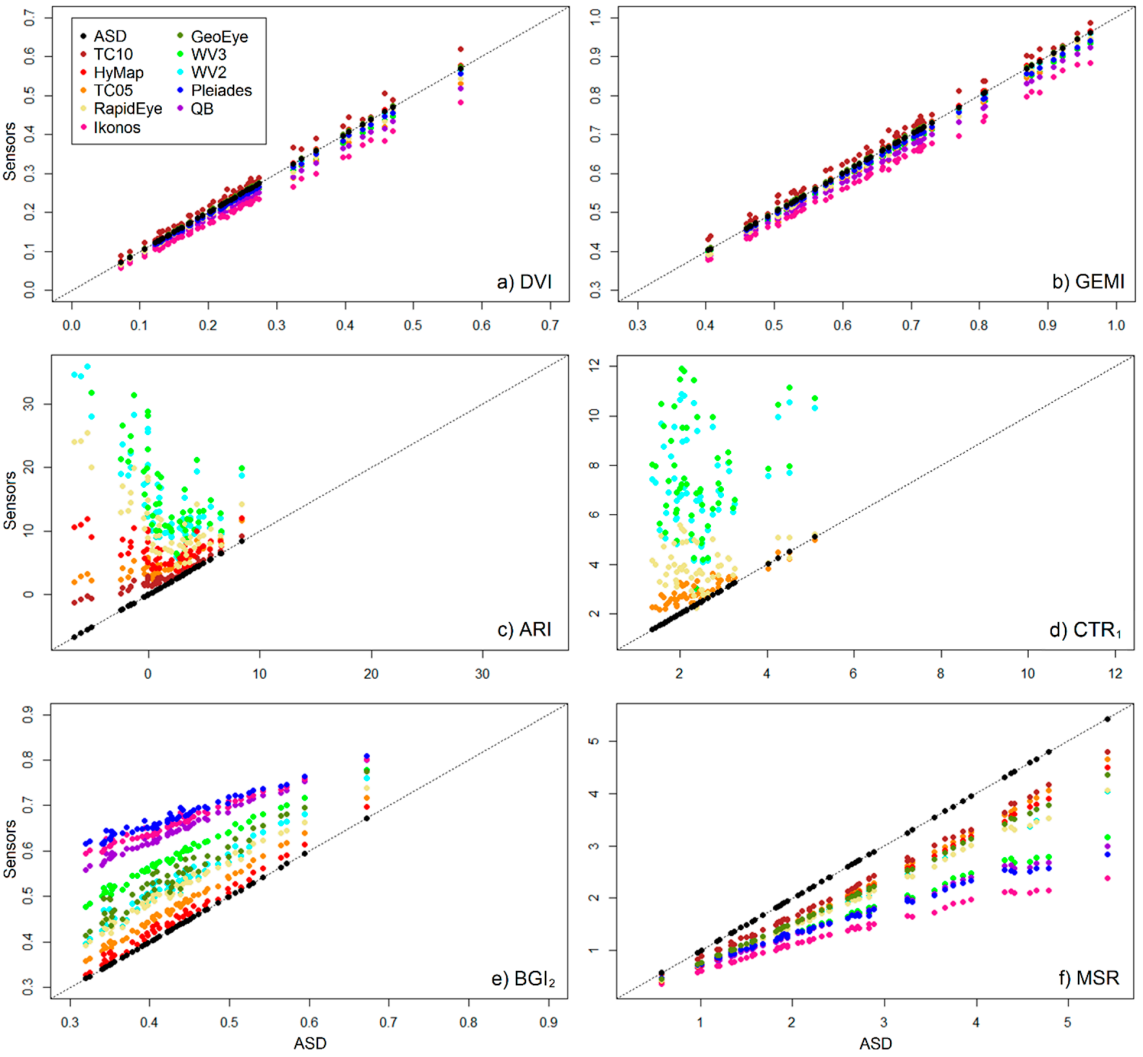
Scatterplots for representative indices, showing the relationships between the original ASD data and the spectrally simulated data of various sensors. (**a**) Difference Vegetation Index (DVI); (**b**) Global Environmental Monitoring Index (GEMI); (**c**) Anthocyanin Reflectance Index (ARI); (**d**) Carter Index 1 (CTR_1_); (**e**) Blue/Green Index 2 (BGI_2_) and (**f**) Modified Simple Ratio (MSR). The dashed line represents the 1:1 line.

The index values for the simulated data of the different sensors were tested against those of the original ASD data and, based on the mean statistical measures, can be grouped into three representative categories: overall performing well (*i.e.*, similar to the original ASD index values), overall performing poorly (*i.e.*, dissimilar to the original ASD index values) and mixed performance. Individual statistical measures for representative indices for these three groups are shown in Table 2, with Difference Vegetation Index (DVI) and Global Environmental Monitoring Index (GEMI) representative of the first group, Anthocyanin Reflectance Index (ARI) and Carter Index 1 (CTR_1_) of the second and Blue/Green Index 2 (BGI_2_) and Modified Simple Ratio (MSR) of the third. Individual statistical measures for all 48 indices are provided in Table S3. For cultivated grasslands, indices that perform well for all measures for the simulated data from all selected sensors compared to the original ASD data are DVI and GEMI (Table 2 and Table S2) as well as Enhanced Vegetation Index (EVI), Modified Chlorophyll Absorption in Reflectance Index 1 (MCARI_1_), Modified Soil-Adjusted Vegetation Index (MSAVI_2_), Modified Triangular Vegetation Index 1 (MTVI_1_), Renormalized Difference Vegetation Index (RDVI), Soil-adjusted Atmospherically Resistant Vegetation Index (SARVI) and Soil-Adjusted Vegetation Index (SAVI, Table S2). This can be observed in the scatterplots for DVI and GEMI (Figure 3a,b), where the points fall close to the 1:1 line with very little scattering, and the slope and intercept are similar to the 1:1 line. All these indices make use of reflectance in the near-infrared and red wavelengths (see Table S1 for index equations). Moreover, the indices all use the difference between the near-infrared and red bands. Most of these indices were originally designed for broad-band sensors. Vegetation’s broad spectral features in the red and near-infrared regions allow these indices to transfer well between sensors of differing band-widths and positions. Despite the good performance of these indices, the relationships between the simulated data and the ASD data do not perfectly fit the 1:1 line (Table 2 and Table S2). Thus for accurate comparisons between index output for data from different sensors, minor adjustments will need to be made using the modeled relationship between the data sets.

For cultivated grasslands, indices that perform the least for all measures for the simulated data compared to the original ASD data are ARI and CTR_1_ (Table 2 and Table S2), as well as Chlorophyll Absorption in Reflectance Index (CARI), Carotenoid Reflectance Index 2 (CRI_700_) and Modified Anthocyanin Reflectance Index (mARI, Table S2). The scatterplots for ARI and CTR_1_ (Figure 3c,d) show general scattering of the points and their deviation from the 1:1 line. For ARI, the slope for some of the simulated data sets (e.g., WorldView-2) even reverses sign, with a resulting large shift in intercept. All these indices include the red-edge band, (Table S1). For green vegetation, there is a sharp order-of-magnitude increase in reflectance in the region of the red-edge (680 nm to 750 nm) [52], over a relatively narrow wavelength range. Thus the position and width of the band in this transitional region will have a considerable effect on the reflectance measured by the sensor (Figure 2). A related and known problem for the cross-calibration of sensors is the spread of the SRFs for the red or near-infrared bands into the red-edge region [2,9,10].

**Table 2:** Individual statistical measures per sensor for representative indices, namely Difference Vegetation Index (DVI) and Global Environmental Monitoring Index (GEMI) representative of the overall performing well group, Anthocyanin Reflectance Index (ARI) and Carter Index 1 (CTR_1_) of the overall performing poorly group and Blue/Green Index 2 (BGI_2_) and Modified Simple Ratio (MSR) of the mixed performance group.

**_1:1_R^2^**										
**Index**	**TC10**	**HyMap**	**TC05**	**RapidEye**	**IKONOS**	**GeoEye**	**WV3**	**WV2**	**Pléiades**	**QB**
ARI	0.576	−3.853	−0.624	−14.216	NA	NA	−34.998	−28.234	NA	NA
BGI_2_	NA	0.944	0.646	−0.260	−8.842	−1.320	−3.378	−0.670	−10.049	−7.247
CTR_1_	NA	NA	0.378	−4.199	NA	NA	−47.760	−40.060	NA	NA
DVI	0.966	0.999	0.983	0.993	0.877	1.000	0.986	1.000	0.995	0.958
GEMI	0.972	1.000	0.986	0.994	0.887	1.000	0.988	1.000	0.995	0.962
MSR	0.860	0.734	0.773	0.613	−0.450	0.716	0.177	0.625	0.039	0.085
**Slope**										
**Index**	**TC10**	**HyMap**	**TC05**	**RapidEye**	**IKONOS**	**GeoEye**	**WV3**	**WV2**	**Pléiades**	**QB**
ARI	0.616	−0.176	0.486	−1.135	NA	NA	−2.000	−1.715	NA	NA
BGI_2_	NA	1.019	1.012	0.953	0.594	0.967	0.848	0.984	0.544	0.693
CTR_1_	NA	NA	0.670	0.186	NA	NA	0.423	0.489	NA	NA
DVI	1.058	1.012	0.959	0.971	0.859	1.009	0.953	0.999	0.974	0.920
GEMI	1.004	1.005	1.001	0.994	0.909	0.998	0.961	0.998	0.965	0.956
MSR	0.868	0.849	0.886	0.757	0.423	0.810	0.567	0.747	0.500	0.542
**Intercept**										
**Index**	**TC10**	**HyMap**	**TC05**	**RapidEye**	**IKONOS**	**GeoEye**	**WV3**	**WV2**	**Pléiades**	**QB**
ARI	2.071	7.105	4.196	12.547	NA	NA	19.477	17.531	NA	NA
BGI_2_	NA	0.008	0.039	0.103	0.406	0.126	0.220	0.102	0.441	0.344
CTR_1_	NA	NA	1.312	3.520	NA	NA	6.547	5.983	NA	NA
DVI	0.003	−0.001	−0.001	−0.001	−0.001	0.000	0.000	−0.001	−0.001	−0.001
GEMI	0.019	−0.001	−0.015	−0.006	0.014	0.004	0.010	0.000	0.014	0.002
MSR	−0.064	−0.179	−0.238	−0.038	0.251	−0.087	0.183	0.003	0.301	0.199
**Normalized Intercept**										
**Index**	**TC10**	**HyMap**	**TC05**	**RapidEye**	**IKONOS**	**GeoEye**	**WV3**	**WV2**	**Pléiades**	**QB**
ARI	1.460	5.010	2.958	8.846	NA	NA	12.361	12.361	NA	NA
BGI_2_	NA	0.018	0.090	0.239	0.942	0.292	0.236	0.236	1.023	0.798
CTR_1_	NA	NA	0.532	1.426	NA	NA	2.425	2.425	NA	NA
DVI	0.012	−0.003	−0.004	−0.004	−0.004	−0.001	−0.004	−0.004	−0.004	−0.004
GEMI	0.029	−0.001	−0.024	−0.010	0.021	0.006	0.000	0.000	0.022	0.003
MSR	−0.026	−0.071	−0.095	−0.015	0.100	−0.035	0.001	0.001	0.120	0.079
**ccR^2^**										
**Index**	**TC10**	**HyMap**	**TC05**	**RapidEye**	**IKONOS**	**GeoEye**	**WV3**	**WV2**	**Pléiades**	**QB**
ARI	0.910	0.067	0.654	0.489	NA	NA	0.556	0.528	NA	NA
BGI_2_	NA	0.991	0.997	0.984	0.978	0.946	0.990	0.962	0.972	0.989
CTR_1_	NA	NA	0.785	0.035	NA	NA	0.024	0.040	NA	NA
DVI	0.994	1.000	0.995	0.999	0.998	1.000	1.000	1.000	1.000	1.000
GEMI	0.995	1.000	0.997	0.999	0.998	1.000	1.000	1.000	1.000	1.000
MSR	0.998	0.999	0.999	0.999	0.989	0.999	0.996	0.999	0.992	0.995

For cultivated grasslands, indices with mixed behavior for the statistical measures comparing the simulated data to the original ASD data are for example BGI_2_ and MSR. They perform well for some sensors and less well for others, while also showing differing performance for the individual measures (Table 2). They show little scatter but their trend lines can deviate quite far from the ideal 1:1 line. The scatterplot of BGI_2_ (Figure 3e) is an example of where both the ccR^2^ and the slope are reasonable but the _1:1_R^2^ and the intercept are poor (Table 2 and Table S2). The scatterplot of MSR (Figure 3f), on the other hand, is an example of where the ccR^2^ and intercept are reasonable but the _1:1_R^2^ and slope are poor (Table 2 and Table S2). Both these scatterplots show instances where the relationship between the simulated sensor data and the original ASD data can be modeled despite their deviation from the 1:1 line. Thus despite the apparent poor _1:1_R^2^, slope and intercept values, many indices may still be transferable across sensors as long as the relationship between the two sensors (for an index) is defined and the ccR^2^ value is close to 1. This is applicable regardless of the spatial resolution of the sensor and is in agreement with numerous studies that found that differences in SRFs cause systematic errors between data from different sensors (e.g., [6,9,10,30]). It should be noted that these relationships may not be linear. For example for the index MSR (Figure 3f), second order polynomial functions model the relationships between the ASD data set and the simulated data sets better than linear functions (with increases in the ccR^2^ values). The literature has examples of where linear translation functions are recommended [1,3,9,12] and where second order polynomial functions are recommended [5,8,10,53]. Additionally, D’Odorico *et al.* [8] found that the choice of regression model is more important than the choice of calibration data source. Worth noting, is that the WorldView-2 and -3 data have the same band widths and positions with only their SRFs differing (WorldView-2 with actual SRFs and WorldView-3 with Gaussian curves), and yet their values for the various measures differ throughout this study (e.g., BGI_2_ in Figure 3, Table 2). It is thus important that actual SRFs be used for spectral cross-calibration and not approximations such as Gaussian curves.

For some indices, no or only weak relationships are apparent (low ccR^2^ values), with much scattering around the trend line (e.g., ARI, Figure 3c). In these cases, these indices are not suitable for cross-calibration between these sensors as there are non-systematic biases resulting from the different SRFs. It should however be noted that the relationships examined in this study are to the narrow-band ASD data. It is possible that for these indices there may be strong relationships between two of the other sensors, particularly if they have similar band widths and positions. Several indices were excluded from this study as they are not appropriate for many sensors since they were designed for narrow spectral features and the bands in the sensors simply either do not cover that region of the electromagnetic spectrum or are too broad. Furthermore, certain indices (particularly those related to moisture) make use of reflectance in the short-wave infrared (SWIR) which is not covered by many sensors and were thus also excluded from the current study. Examples are the Photochemical Reflectance Index (PRI) which uses reflectance at 528 nm and 567 nm [54] and the Normalized Difference Water Index (NDWI) which uses reflectance at 860 nm and 1240 nm [55].

When applied to images, accounting for other parameters such as atmospheric variables, solar and observational geometries, spatial resolution, *etc.* [3,7,10] is necessary to achieve high cross-calibration accuracy. The effect that different spatial resolutions of various sensors have on NDVI and other index values depends on the nature (and spatial extent) of the target [4]. A related, but often neglected, consideration is the effects of the change in support (scale effect) between sensors which affects items such as the variance [56] and range [57] of values measured by various sensors. It has been recommended [10,30] that step-wise correction be applied for cross-calibration between sensors, with SRF effects being corrected first followed by correcting for residual factors such as atmospheric conditions, sun- and viewing angles and spatial resolution.

### 3.2. Correlation to Quality Indicators

#### 3.2.1. Soil Moisture

Cundill *et al.* [21] show that several spectral indices correlate with the soil moisture quality indicator for grass covered dikes, where they function as proxies for the vegetation’s response to available soil moisture. In the current paper, indices which have the highest correlations to soil moisture across all sensors are Ratio Vegetation Index (RVI), Green/Red Ratio (GRR), MSR, Gitelson and Merzlyak 2 (GM_2_), and Modified Simple Red Edge Ratio Index (mSR_705_, mean |r| > 0.550, Table S4). These results are similar to what Cundill *et al.* [21] found, where Ratio Vegetation Index 1 (RVI_1_), Near-infrared/Red Ratio (NIRRR), RVI (which are all variants of near-infrared:red ratio) and a broad-band green:red ratio have the highest correlations to soil moisture. GRR is equivalent to the broad-band green:red ratio and RVI and MSR are variants of the ratio near-infrared:red. Indices that were identified in the previous section as transferring particularly well between sensors (*i.e.*, DVI, EVI, GEMI, MCARI_1_, MSAVI_2_, MTVI_1_, RDVI, SARVI and SAVI, see Table S2), but which all make use of the difference between the near-infrared and red bands, do not have high correlation coefficients for soil moisture (mean |r| < 0.210; Table S4). GM_2_ and mSR_705_ make use of near-infrared:red-edge ratios. This appears contrary to what was discussed in the previous section, where the use of the transitional red-edge region indicated that an index would not transfer well. However, indices ARI, CARI, CRI_700_, CTR_1_ and mARI (which make use of the red-edge band and have low ccR^2^ values) primarily use a form of the difference between the red-edge band and the green band (Table S1), with the exception of CTR_1_ (which uses a red-edge:blue ratio). It would thus seem that indices that ratio the near-infrared and red-edge bands can be used for correlation with soil moisture of grass covered surfaces. Further, it would also appear that for correlation to soil moisture, the proportional (ratio) reflectance of bands is important for adjusting for the effects of SRFs on indices rather than the difference in reflectance between bands.

This indeed would seem to be true. When examining vegetation spectra, there is an order-of-magnitude increase from red to near-infrared. Decreasing plant water content causes physiological changes that result in an increase in the near-infrared reflectance and a lesser increase in the red reflectance [58,59]. However, proportionally the increase in the red reflectance is larger because of relatively low absolute reflectance values. This explains why the RVI and MSR indices, and also the GM_2_ and mSR_705_ indices, work well for soil moisture of grass covered surfaces while the DVI, EVI, GEMI, MCARI_1_, MSAVI_2_, MTVI_1_, RDVI, SARVI and SAVI indices do not (Table S4). For the GRR index, decreasing plant water content results in an overall increase in reflectance in the visible wavelengths (400–700 nm) due to leaf pigments’ physiological dependence on water [43,60], with a greater proportional increase in red reflectance than in green (as observed in Figure 2 of [60]). Positive correlations were also found between GRR and water potential in a study by Rodriguez *et al.* [43] on grapevines. Further, with extreme water deficiencies, leaves turn yellow or brown (leaf firing) and ultimately result in an increase in standing litter (dead vegetation). Standing litter affects reflectance in that, similar to water deficit stress, there is an overall increase in reflectance in the visible spectrum, with a greater proportional increase in red than in green reflectance, although with much larger increases in magnitude (as observed in Figure 8 of [61]). Thus, for the current study, where there is also dead vegetation (most likely due to water deficit), the GRR index also correlates relatively well with soil moisture. Overall, the narrow-band sensors appeared to perform better than the broad-band sensors for the five indices with the highest correlations to the soil moisture indicator (Table S4). However, the broad-band RapidEye, GeoEye-1 and WorldView-2 sensors also performed relatively well.

#### 3.2.2. Cover Quality

Cundill *et al.* [21] found that several spectral indices correlate with the dike cover quality indicator. In the current paper, indices Red Edge Normalized Difference Vegetation Index (NDVI_705_), Modified Red Edge Normalized Difference Vegetation (mNDVI_705_), mSR_705_, GM_2_, Carter Index 2 (CTR_2_), Red/Green Index (RGI), GRR and Normalized Green/Red Ratio (NGRR) have the highest correlations to cover quality across all the sensors (mean |r| > 0.580, Table S5). Indices NDVI_705_, mNDVI_705_, mSR_705_, GM_2_ and CTR_2_ use near-infrared and red-edge bands and the remaining three (RGI, GRR and NGRR) use green and red bands. These indices use either the ratio between the bands (*i.e.*, mSR_705_, GM_2_, CTR_2_, RGI and GRR) or the difference between the bands (*i.e.*, NDVI_705_, mNDVI_705_ and NGRR). Cover quality for the dike is determined by the presence and proportions of healthy grass, dry grass (standing litter) and bare soil. Dense green grass cover, with high LAI values, affect the reflectance spectra in that there is an order-of-magnitude increase in the near-infrared reflectance, an increase in the red-edge, a decrease in the red reflectance and little change in the green (as observed in Figure 5 of [61]). The presence of standing litter and bare soil affect the whole spectrum but non-linearly, with, for example, larger increases in reflectance in the red than the green (as observed in Figures 2, 3, 8 and 9 of [61]). The combined effects of the amount of lush green vegetation, standing litter and bare soil on reflectance are such that relationships to cover quality can be established using indices NDVI_705_, mNDVI_705_, mSR_705_, GM_2_, CTR_2_, RGI, GRR and NGRR.

Although these indices were not those found by Cundill *et al.* [21] to have the highest correlations for the original ASD data (of which the highest is 0.630 for an index not valid for this study), their values still range between ±0.567 and ±0.580 for this data set (Table S5). Indices that were identified in the previous section as transferring particularly well between sensors (*i.e.*, DVI, EVI, GEMI, MCARI_1_, MSAVI_2_, MTVI_1_, RDVI, SARVI and SAVI, see Table S2), all make use of the difference between the near-infrared and red bands and do not have high correlation coefficients for cover quality (mean |r| < 0.205, Table S5). However, some indices that make use of the near-infrared and red bands (e.g., NDVI, RVI) have correlations for the original ASD data in the range of ±0.576 and ±0.583, with mean |r| values in the range of 0.578 and 0.580 across all sensors (Table S5). These include indices that ratio the near-infrared bands as well as those that difference them. Thus correlation to cover quality depends not only on the bands used by the index but also the computation of the index, which cannot be simply separated based on ratio or difference.

Although the indices that performed well for cover quality were designed for narrow-band sensors, the spectral features for cover quality in the green, red and near-infrared are broad. Thus for an application such as grass cover quality assessment, broad-band sensors can perform as well and sometimes better than narrow-band sensors. This is in agreement with other studies which found that for some applications broad-band indices perform almost as well or better than the equivalent narrow-band indices [48], particularly if attention is paid to the position of the bands [62] and the parameter which is being estimated [63]. This is favorable for the implementation of operational systems for monitoring and assessing cultivated grasslands, including dike inspection and monitoring, because broad-band satellite data are cheaper and more easily available than airborne and narrow-band data.

## 4. Conclusions

This paper examined the effects of differing spectral response functions (SRFs) on the cross-calibration of a large number of indices across various very high spatial resolution sensors for cultivated grasslands. The data for the various sensors were simulated by convolving field reflectance spectra using sensor specific SRFs, which enabled the investigation of the SRF effects alone without being affected by other factors such as atmospheric condition or spatial resolution. Index values calculated from data of sensors with differing spectral response functions are not directly comparable. Broad-band indices DVI, GEMI, EVI, MSAVI_2_ and SAVI render the most similar values per index across all sensors tested (1:1 line mean _1:1_R^2^ > 0.960 and linear trend mean ccR^2^ > 0.997), but despite their similarity, there are differences which should be adjusted for when comparing absolute index values between sensors. For many indices, the index values altered significantly with the changing spectral resolution of the sensors. However, in most cases, relationships could be established between the index values for different sensors and the narrow-band ASD data. The width and position of bands that fall within transitional regions of the spectrum (e.g., red-edge) adversely affect the relationships for indices that use these bands. The correct definition of the relationship is necessary for accurate adjustment of SRF effects and is often linear or second order polynomial. Thus adjusting for the effects of SRFs on indices between sensors of different spectral resolutions is possible as long as the relationship of the index values between these sensors can be modeled and the square of the correlation coefficient (ccR^2^) is close to 1. It is crucial that the full definition of the SRFs be used and not only the band widths and positions. It is recommended that when applied to indices obtained from images of different sensors, not only SRF effects should be considered but also the effects of items such as atmospheric conditions, solar and observational geometries, spatial resolution and change in support.

The performance of indices across sensors was tested for an application. Correlations to two indicators were tested in the context of inspection and monitoring of grass covered dikes, although the results could be more generally applied to cultivated grasslands. For the soil moisture indicator, indices that ratio bands performed better across sensors than those that difference bands, with ratios using the near-infrared and red bands out-performing others. Although the index values were different between sensors, the correlation coefficients were similar because the relationships between the sensors could be defined. Similarly, various indices had similar correlation coefficients across different sensors to the indicator of dike cover quality. However, for this indicator, the bands used by the index affect the performance as well as how they are applied. The results show that for certain indices, sensors with broad spectral bands can perform almost as well or better than narrow-band sensors for the estimation of these parameters and that for many indices it is not necessary to have narrow-band data if the appropriate bands are defined in the broad-band system.
